# In comparative analysis of multi-kinase inhibitors for targeted medulloblastoma therapy pazopanib exhibits promising *in vitro* and *in vivo* efficacy

**DOI:** 10.18632/oncotarget.2240

**Published:** 2014-07-18

**Authors:** Rogerio B. Craveiro, Michael Ehrhardt, Martin I. Holst, Thorsten Pietsch, Dagmar Dilloo

**Affiliations:** ^1^ Department of Pediatric Hematology and Oncology, Center for Pediatrics, University of Bonn Medical Center, Adenauerallee 119, D-53113 Bonn, Germany; ^2^ Department of Neuropathology, University of Bonn, Sigmund-Freud-Str. 25, 53105 Bonn, Germany

**Keywords:** Medulloblastoma, Sorafenib, Pazopanib, Targeted therapy, Multi-kinase inhibitor (MKI)

## Abstract

Regardless of the recent advances in cytotoxic therapies, 30% of children diagnosed with medulloblastoma. succumb to the disease. Therefore, novel therapeutic approaches are warranted. Here we demonstrate that Pazopanib a clinically approved multi-kinase angiogenesis inhibitor (MKI) inhibits proliferation and apoptosis in medulloblastoma cell lines. Moreover, Pazopanib profoundly attenuates medulloblastoma cell migration, a prerequisite for tumor invasion and metastasis. In keeping with the observed anti-neoplastic activity of Pazopanib, we also delineate reduced phosphorylation of the STAT3 protein, a key regulator of medulloblastoma proliferation and cell survival. Finally, we document profound *in vivo* activity of Pazopanib in an orthotopic mouse model of the most aggressive *c-myc* amplified human medulloblastoma variant. Pazopanib reduced the growth rate of intracranial growing medulloblastoma and significantly prolonged the survival. Furthermore, to put these results into a broader perspective we analysed Pazopanib side by side with the MKI Sorafenib. Both compounds share a similar target profile but display different pharmacodynamics and pharmacokinetics with distinct cytotoxic activity in different tumor entities. Thus, we identified Pazopanib as a new promising candidate for a rational clinical assessment for targeted paediatric medulloblastoma therapy.

## INTRODUCTION

Medulloblastoma is the most common malignant paediatric brain tumor, with 85% of medullolastoma being diagnosed in patients younger than 18 years of age. Regardless of the recent advances in clinical risk stratification and cytotoxic therapies, 30% of children succumb to the disease [1]. Survivors often suffer from long-term side effects of chemotherapy [2]. Therefore incorporating biologically targeted therapy into treatment strategies for medulloblastoma is of considerable interest.

Based on transcriptomic approaches the current consensus is that medulloblastoma is comprised of four distinct molecular subgroups - SHH, WNT, group 3 and group 4 – which correspond in part to the former histological WHO classes for medulloblastoma [3-4]. Common to all molecular subgroups is the deregulation of receptor tyrosine kinases such as members of the platelet-derived growth factor receptor (PDGFR) family, the vascular endothelial growth factor receptor (VEGFR) family and the tyrosine kinase c-kit. Indeed, activation of these receptor tyrosine kinases is considered a hallmark of medulloblastoma development and progression [3, 5-6]. Also, non-receptor tyrosine kinases such as the SRC-family kinases and serine/threonine specific kinases including aurora A and maternal embryonic leucine zipper kinase (MELK) have been implicated in medulloblastoma formation [7-9].

Pazopanib represents a new addition to the multi-kinase inhibitors (MKI) that target tumor angiogenesis via inhibition of the VEGFR pathway [10]. Pazopanib has recently been approved for the treatment of patients with renal cell carcinoma and soft tissue sarcoma and has also shown good activity and tolerability in ovarian, cervical and early stage non-small lung cancer [10-14]. Emerging data indicate that Pazopanib similar to the MKI Sorafenib can penetrate the blood brain barrier, which suggests that these drugs may serve as useful therapeutics for medulloblastoma treatment [15-17]. However, clinical data on MKI treatment in pediatric brain tumors are sparse with no reports on the efficacy of Pazopanib for medulloblastoma therapy and only one *in vitro* study documenting Sorafenib's capacity to target medulloblastoma [18-19].

The anti-neoplastic activity of Pazopanib and Sorafenib is not only based on inhibition of tumor angiogensis by targeting blood vessel formation but is also due to blockade of oncogenic kinases in the neoplastic cells themselves. Analysis of the target profile of Pazopanib and Sorafenib revealed that both inhibitors target the key drivers of medulloblastoma development mentioned above. Yet, although the multi-kinase inhibitors share many targets, the exact target composition and affinities are unique to each compound [20]. MKI also display diverse pharmacokinetics and thus vary in bioavailability [21-22]. It is these differences that account for the differential efficacy in neoplastic disease and distinct toxicity profiles [23-24]. Furthermore, MKI-mediated suppression of the hematopoietic and immune system is a critical aspect when considering administration of these drugs in combination with myelosuppressive chemotherapy for enhanced potency [20]. Therefore the objective of our study was to evaluate Pazopanib and Sorafenib for targeted medulloblastoma therapy *in vitro* and *in vivo*.

Here, we demonstrate for the first time that Pazopanib profoundly inhibits proliferation and induces significant apoptosis in a spectrum of paediatric medulloblastoma cell lines. Furthermore, we show that Pazopanib attenuates medulloblastoma cell migration a pre-requirement for invasion and that these anti-carcinogenic effects are associated with markedly suppressed phosphorylation of the signal transducer and activator of transcription 3 (STAT3) protein. Furthermore, we present first evidence that the in vitro activity of Pazopanib and Sorafenib translates into profound anti-neoplastic efficacy in an orthotopic xenograft mouse model of the most aggressive *c-myc* amplified medulloblastoma variant. Pazopanib and Sorafenib decelerate tumor growth and significantly prolong the survival of mice bearing intracranial human medulloblastoma. Analysing Pazopanib side by side with the MKI Sorafenib shows that both compounds display a similar anti-carcinogenic capacity *in vitro* and *in vivo*. Based on its favourable cytotoxicity profile recommends itself Pazopanib in combination with standard treatment regimes for targeted medulloblastoma therapy.

## RESULTS

### In medulloblastoma Pazopanib and Sorafenib treatment leads to dose-dependent reduction of cell viability

**Fig 1:**
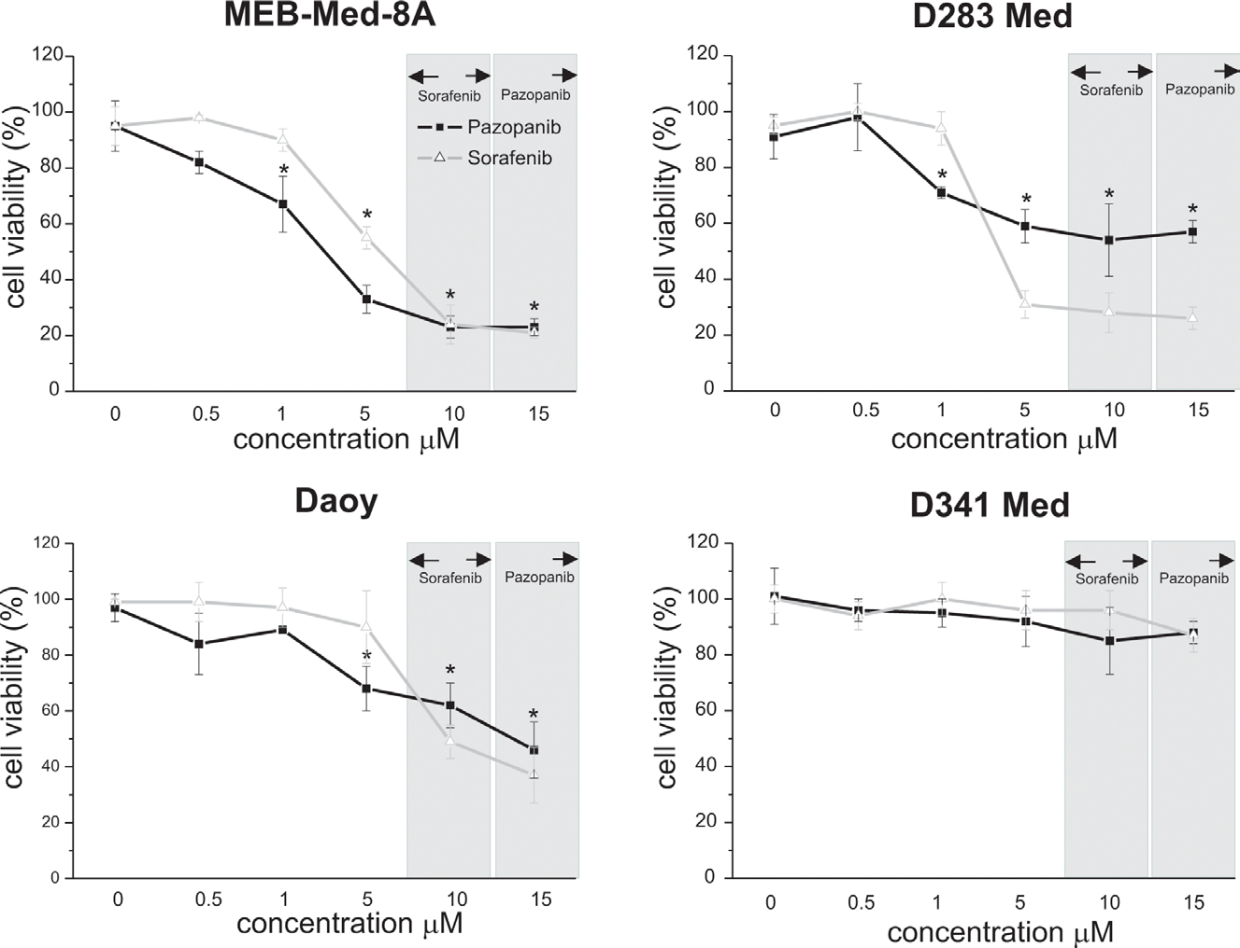
In medulloblastoma Pazopanib and Sorafenib treatment leads to a dose-dependent reduction of cell viability The cell lines MEB-Med-8A, D283 Med, Daoy and D341 Med were treated with increasing concentrations of Pazopanib and Sorafenib. Areas shaded in grey indicate the range of the respective MKI concentrations detectable in patient's plasma. The vehicle DMSO served as control. After 48h of drug exposure the cell viability was assessed by MTS assay. Values below an asterisk are significantly different from the control (*p<0,05). Each experiment was performed in triplicates and repeated four times.

In a dose-response study the cytotoxic potency of Pazopanib and Sorafenib was assessed in the medulloblastoma cell lines Daoy, MEB-Med-8A, D283 Med and D341 Med (Figure 1). For comparative assessment of cell viability by MTS assay, MKI concentrations were chosen to reflect the range of different plasma levels observed in patients for MKIs namely 10 μM for Sorafenib and 15 μM for Pazopanib [21-22]. The vehicle DMSO served as control. At the lowest concentration of 0.5 μM, all 4 medulloblastoma cell lines proved itself entirely MKI-resistant but responded significantly to higher MKI concentrations. At 15 μM Pazopanib and 10 μM Sorafenib showed comparable efficacy in 3 of 4 medulloblastoma cell lines as documented by low level residual cell viability (Pazopanib: MEB-Med-8A 22.5±3.3%, D283 Med 56.7±4%, Daoy 45.7±10.2%; Sorafenib: MEB-Med-8A 21.1±1.7%, D283 Med 26±3.5%, Daoy 36.8±9.9%). When exposing D341 Med to the same concentration of Pazopanib and Sorafenib only a marginal growth suppressive effect was observed (Pazopanib: D341 Med 88±4.2%; Sorafenib: D341 Med 86.5±6.3%)

### Pazopanib and Sorafenib exert anti-proliferative and pro-apoptotic effects in medulloblastoma cell lines

At concentrations corresponding to patient plasma levels we further dissected the kinetics of the anti-proliferative and pro-apoptotic effects of MKI treatment over an extended incubation period of up to 72h by means of a flow cytometry-based proliferation/apoptosis assay [21-22].

**Fig 2:**
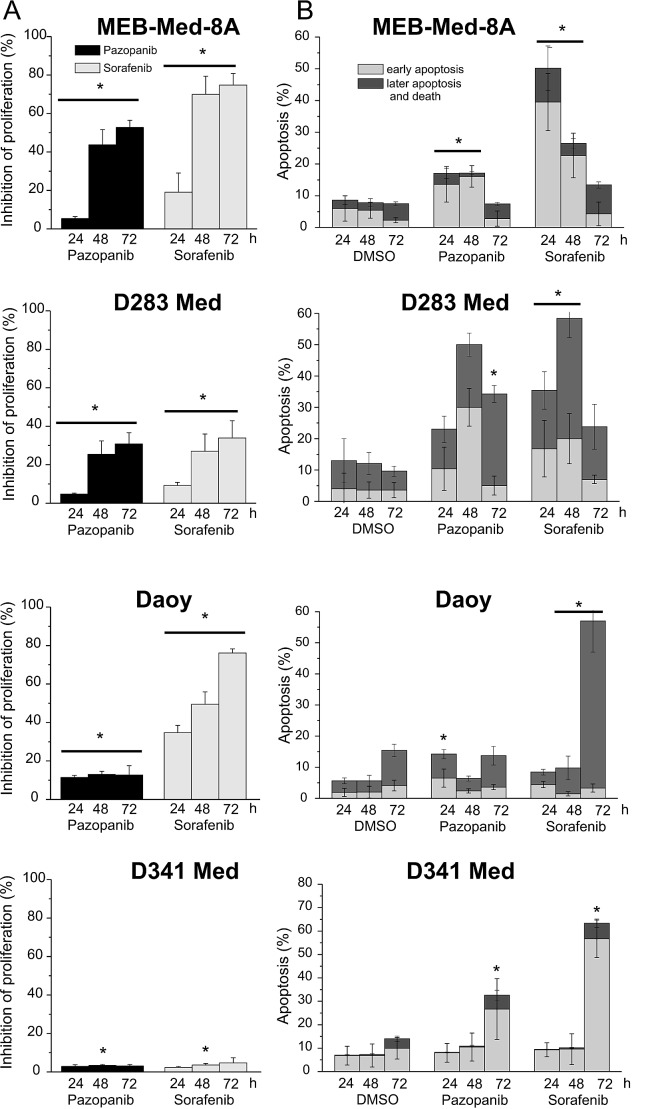
Pazopanib and Sorafenib exhibit anti-proliferative and pro-apoptotic effects in medulloblastoma cell lines In a combined proliferation-apoptosis assay based on a CFSE-7AAD-Annexin-V staining the capacity of Pazopanib and Sorafenib to inhibit proliferation (a) and induce apoptosis (b) in medulloblastoma cell lines was determined. The cells were treated for 24, 48 and 72h with MKI concentrations corresponding to patient's plasma levels (Pazopanib 15 μM and Sorafenib 10 μM). The vehicle DMSO served as control. The proliferation were normalized with the DMSO control. Statistically significant differences compared to control are marked by an asterisk (*p<0,05). The data represent four independent experiments.

After 72h treatment significant suppression of cellular growth was observed in 3 of 4 investigated medulloblastoma cell lines (Figure 2a). Pazopanib and Sorafenib inhibited proliferation in MEB-Med-8A (Pazopanib: 52.8±3.6%, Sorafenib: 74.8±6%) and D283 Med (Pazopanib: 30.8±5.9%; Sorafenib: 33.9±9.2%) to a similar extent. In Daoy the suppression effect of Sorafenib was more pronounced (Pazopanib: 12.7±4.8%; Sorafenib: 76±2.2%), while D341 proved resistant to both compounds.

Pazopanib and Sorafenib induced apoptosis in all investigated medulloblastoma cell lines (Figure 2b). Sorafenib exhibited a strong pro-apoptotic activity with maximum apoptosis rates of 50-70%, albeit at different time-points for each cell line (MEB-Med-8A at 24h 50.3±12%, D283 Med at 48h: 53.4±15%, Daoy at 72h: 57±10%; D341 Med 72h: 73.6±15%). Pro-apoptotic activity of Pazopanib also peaked at variable time-points for each cell line with maximum apoptosis rates of 20-40% (MEB-Med-8A at 48h 16.9±3.3%, D283 Med at 48h 29.6±8.6, Daoy at 24h 14.1±8.5%, D341 Med at 72 h 42.7±12%).

### Pazopanib and Sorafenib induce S-phase cell cycle arrest

**Fig 3:**
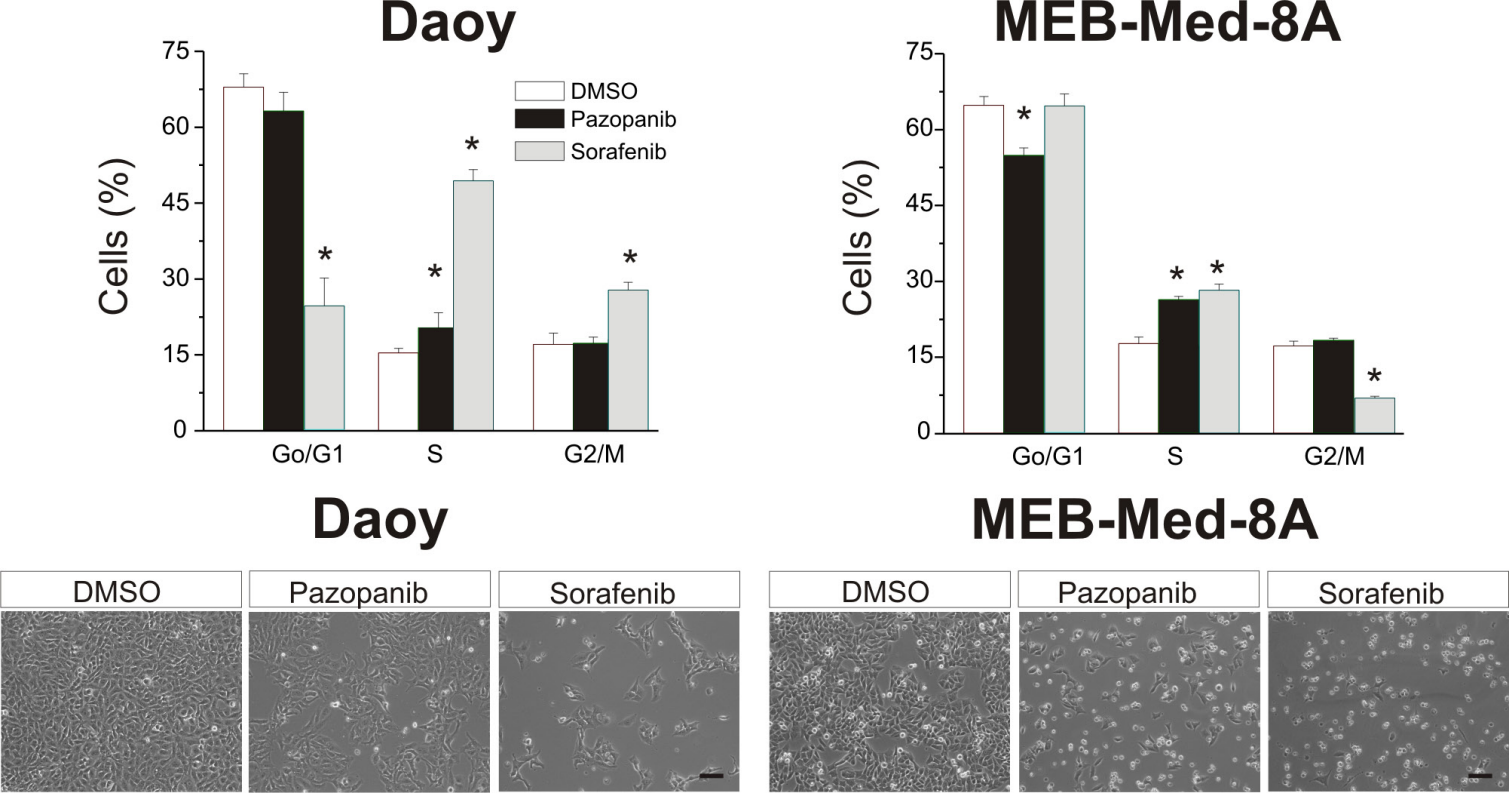
Pazopanib and Sorafenib induce S-phase cell cycle arrest Daoy and MEB-Med8A cells were treated for 48h with concentrations corresponding to patient's plasma levels (Pazopanib 15 μM and Sorafenib 10 μM). Subsequently cell cycle distribution was determined by Hoechst 33342 staining. The vehicle DMSO served as control. The lower panel depicts the reduction in cell density and changes in morphology for Daoy and MEB-Med-8a after drug exposure for 48h (scale bar 100 μm). Statistically significant differences from control are marked by an asterix (*p<0,05). The data shown represent five independent experiments.

MEB-Med-8A is of higher and Daoy of lower sensitivity to Pazopanib-mediated anti-proliferative effects, therefore these cell lines were chosen for cell cycle analysis after 48h exposure to the respective MKI. The vehicle DMSO served as control (Figure 3). MKI were applied at concentrations corresponding to patient plasma levels [21-22]. Treatment with either Pazopanib or Sorafenib induced a significant S-Phase arrest in MEB-Med-8A (Pazopanib 26.4±1.6%; Sorafenib 28.2±1.2%; DMSO 17.7±1.3%) and Daoy (Pazopanib 20.3±2.9%; Sorafenib 49.4±2.2%; DMSO 15.3±0.9%). Moreover, for Daoy an additional G_2_M-Phase arrest was detected in the presence of Sorafenib (Sorafenib 27.8±1.5% and DMSO 17.0±2.2%).

### Pazopanib and Sorafenib impair colony formation of medulloblastoma cells

**Fig 4:**
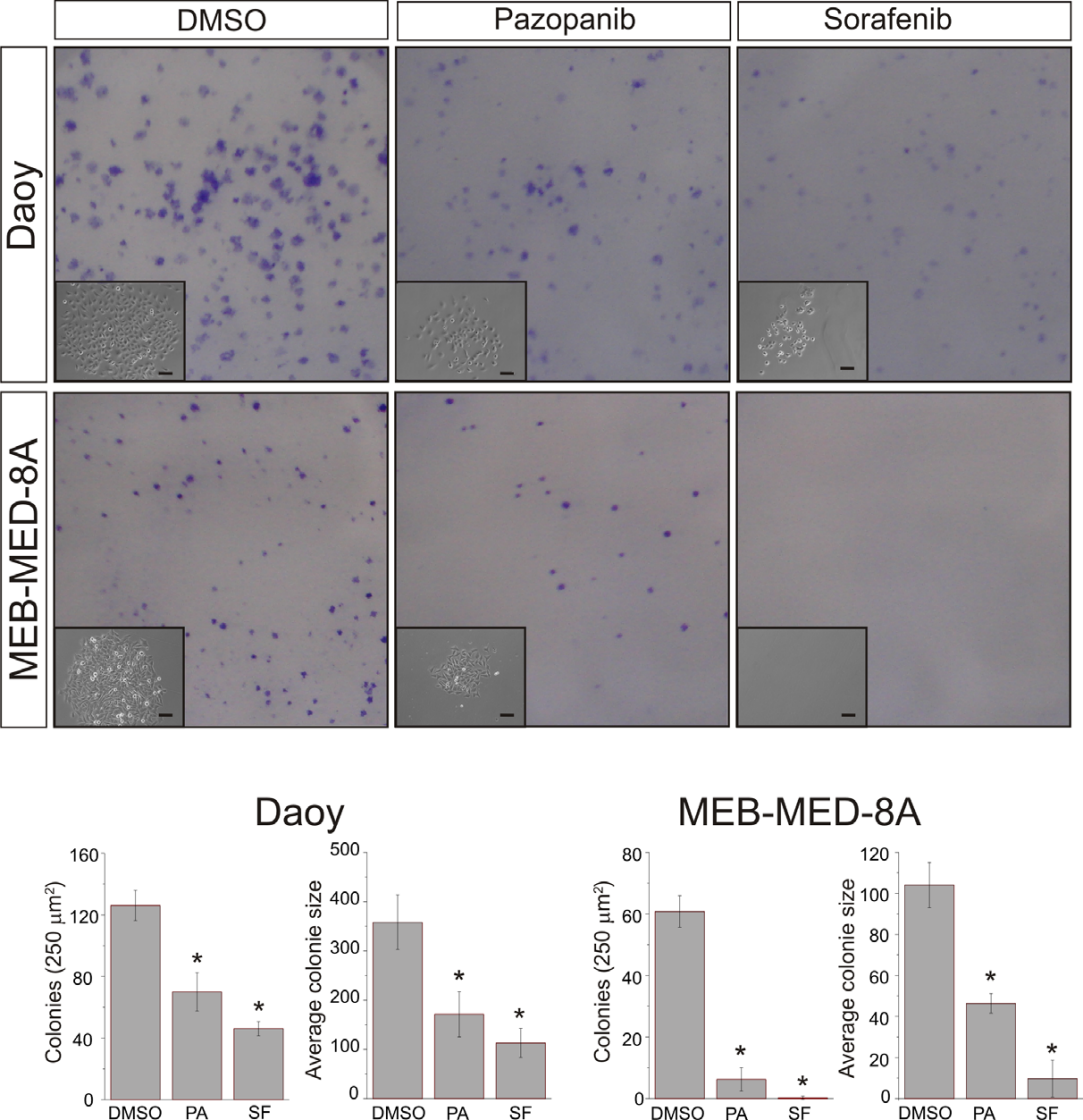
Pazopanib and Sorafenib impair colony formation of medulloblastoma cells Daoy and MEB-Med-8A cells were exposed to 15 μM of Pazopanib and 10 μM of Sorafenib respectively for 48h. Subsequently the cells were maintained in standard growth medium for 7 days and colony formation and colony size were assessed. Statistically significant differences are marked by an asterisk (*p<0.05). The data shown represent five independent experiments.

We also analyzed the capability of Pazopanib and Sorafenib to interfere with clonogenicity of the adherent cell lines MEB-Med-8A and Daoy (Figure 4). For this purpose MEB-Med-8A and Daoy and were exposed to clinically relevant concentrations of Pazopanib and Sorafenib respectively for 48h. Thereafter the cells were cultured for 7 days in standard medium without drugs. The number of colonies (NC) and the average colonies size (ACS) were determined.

Treatment of MEB-Med-8A with 15 μM Pazopanib and 10 μM Sorafenib respectively resulted in a significant reduction of colony numbers (Pazopanib 6.2±3.8, Sorafenib 0.25±0.5, DMSO 60.7±5.1) and average colony size (Pazopanib 46.4±4.8p^2^, Sorafenib 9.7±9p^2^, DMSO 104±11p^2^). A similar result was observed when exposing Daoy to the same concentrations of Pazopanib and Sorafenib both compounds inhibited colony formation profoundly (Pazopanib 70±12.4, Sorafenib 46±4.7, DMSO 126±9.9) and reduced the size of growing colonies (Pazopanib 171±46p^2^, Sorafenib 113±29.5p^2^ DMSO 358±55p^2^).

### The anti-proliferative and pro-apoptotic effects of Pazopanib and Sorafenib are associated with a reduction of STAT3 phosphorylation at tyrosine 705

**Fig 5:**
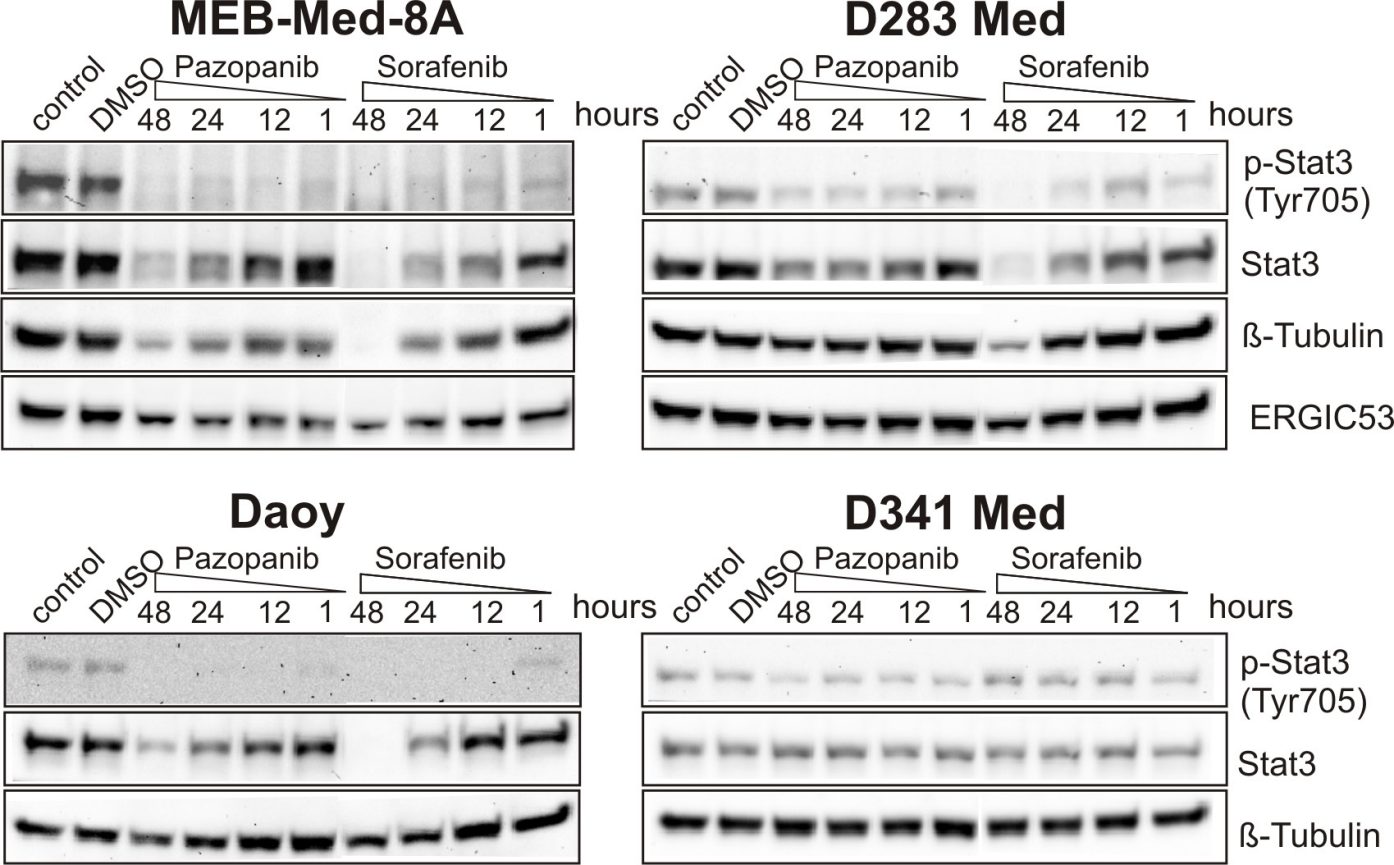
The anti-proliferative and pro-apoptotic effects of Pazopanib and Sorafenib are associated with a reduction of STAT3 phosphorylation at tyrosine 705 In Daoy, MEB-Med-8A, D283 Med and D341 Med cells were treated with Pazopanib and Sorafenib at concentrations corresponding to patient's plasma levels (Pazopanib 15 μM and Sorafenib 10 μM) for a 1, 12, 24 and 48h period. Total protein levels and the phosphorylation status of STAT3 were determined by westernblot. Beta-tubulin and ERGIC53 respectively served as loading controls. The data-set shown represents 1 of 3 independent experiments.

In keeping with previous reports documenting constitutive STAT3 expression and phosphorylation in human medulloblastoma biopsies, all 4 investigated medulloblastoma cell lines exhibited STAT3 phosphorylation at TYR705. Here we document for the first time that Pazopanib similar to Sorafenib reduced STAT3 phosphorylation in 3 of 4 medulloblastoma cell lines in a dose- (data not shown) and time-dependent manner (Figure 5). At clinically relevant concentrations Pazopanib and Sorafenib profoundly inhibit STAT3 phosphorylation in MEB-Med-8A, D283 Med and Daoy, but not in the most resistant cell line D341 Med [21-22].

In addition to blockade of STAT3 phosphorylation, in MEB-Med-8A, D283 Med and Daoy, after prolonged Pazopanib and Sorafenib exposure STAT3 protein synthesis itself was profoundly suppressed (Figure 5 48h). Furthermore, in the most drug sensitive cell line, MEB-Med -8A, long-term treatment with Pazopanib and Sorafenib led to a decrease of beta-tubulin protein levels in contrast to stable levels of the house-keeping protein ERGIC53. These effects were not observed in the cell line D341 Med.

### Pazopanib and Sorafenib inhibit medulloblastoma cell migration

**Fig 6:**
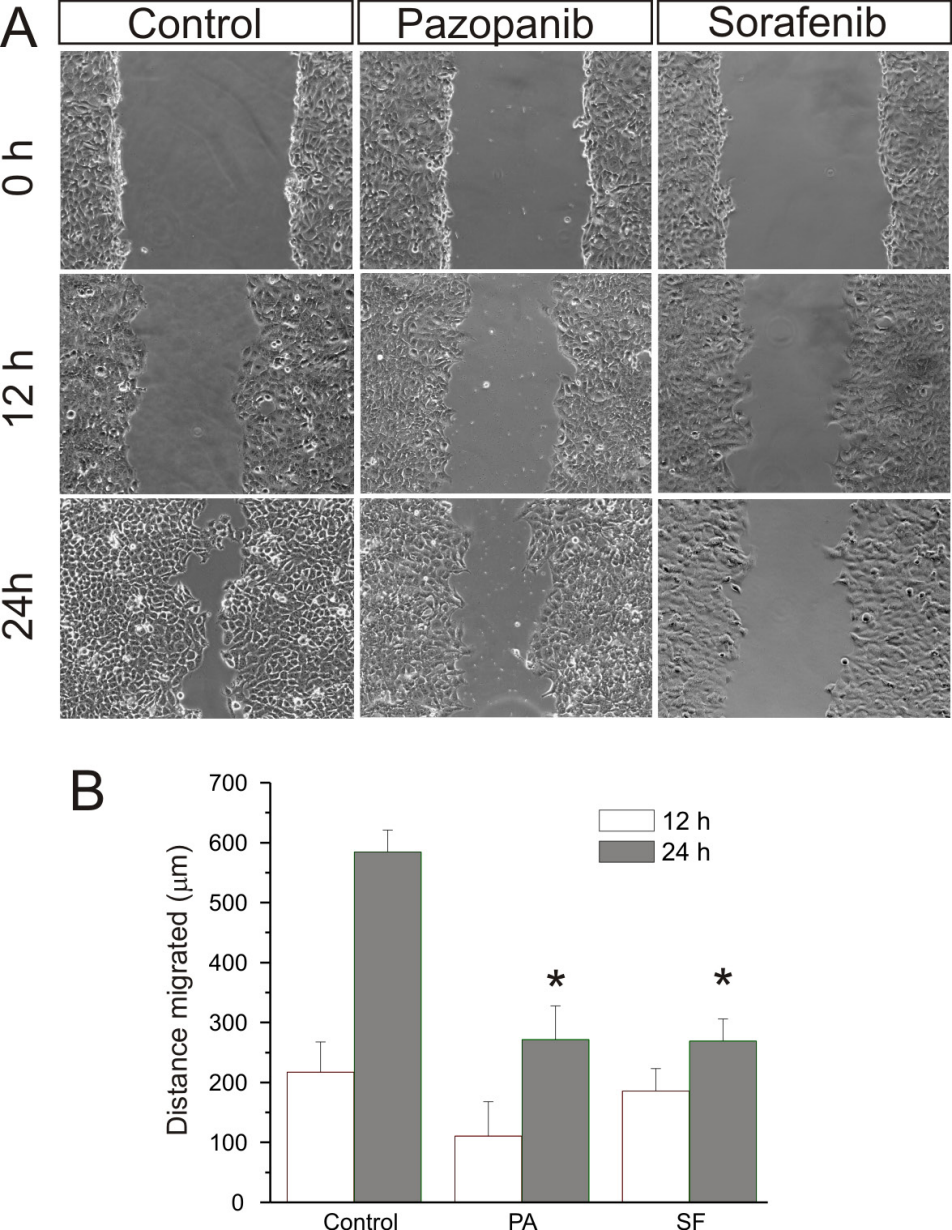
Pazopanib and Sorafenib inhibit medulloblastoma cell migration After a single scratch was made in a confluent monolayer of Daoy cells, these were exposed to Pazopanib and Sorafenib at concentrations corresponding to patient plasma levels (Pazopanib 15 μM and Sorafenib 10 μM) for 24h. Each scratch was photographed after 12 and 24h and its width determined. Statistically significant differences from control are marked by an asterisk (*p<0,05). The data shown represent four independent experiments.

In migration assays we determined the potential of Pazopanib and Sorafenib to inhibit medulloblastoma cell migration, a prerequisite for invasion and metastasis (Figure 6a and 6b). We chose the medulloblastoma cell line Daoy based on its known migratory properties. At concentrations comparable to those observed in patient plasma, Pazopanib and Sorafenib significantly inhibited cell migration in a scratch wound assay in comparison to the vehicle DMSO employed for control. (Migration at 24h: Pazopanib 271 ± 56 μm; Sorafenib 269 ± 37μm and DMSO 584 ± 36 μm) [21-22].

### Pazopanib and Sorafenib delay tumor growth *in vivo* and prolong the survival of mice bearing intracranial human medulloblastoma

**Fig 7:**
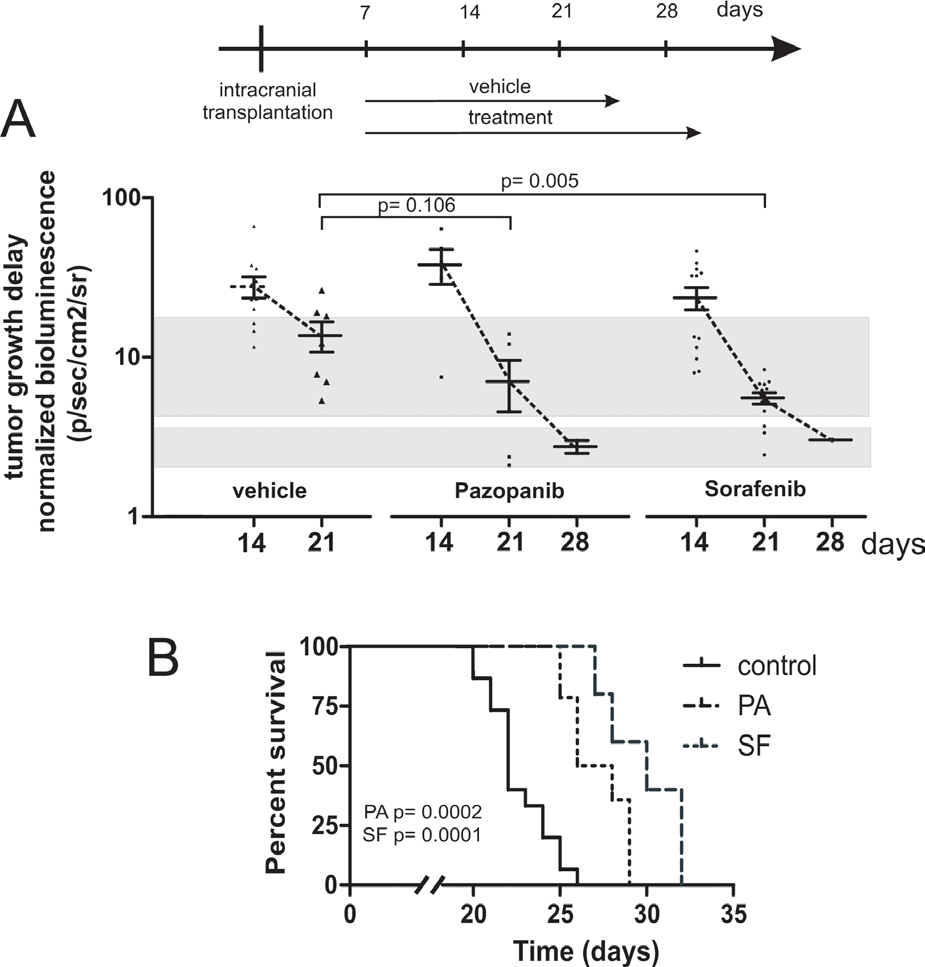
Pazopanib and Sorafenib delay tumor growth *in vivo* and prolong the survival of mice bearing intracranial human medulloblastoma In a orthotopic xenograft mouse model we analysed whether Pazopanib and Sorafenib could inhibit medulloblastoma growth *in vivo*. For this purpose 2×10^4^ MEB-Med-8A cells were transplanted into the cerebellum of mice to establish tumors. The tumor growth was analyzed by bioluminescent imagining after 1, 2, 3 and 4 weeks. One week after transplantation mice were treated with 60 mg/kg of Pazopanib and 30 mg/kg of Sorafenib once daily by gavage until they developed symptoms. Figure 7a depicts the normalized tumor growth delay while figure 7b shows the survival of treated and untreated animals via Kaplan-Meyer curve. Pazopanib and Sorafenib treatment prolonged the survival of medulloblastoma bearing mice significantly.

In an orthotopic mouse model we analyzed the capacity of Pazopanib and Sorafenib to inhibit human medulloblastoma growth *in vivo* (Figure 7). For this purpose 2×10^4^ lentivirally transduced MEB-Med-8A cells stably expressing luciferase were transplanted into the cerebellum of immunocompromised mice resulting in reliable tumor formation as early as one week post transplantation. Animals with established tumors were treated with 60 mg/kg of Pazopanib and 30 mg/kg of Sorafenib respectively. Tumor growth was monitored via bioluminescent imagining and mice showing clinical impairment due to tumor progression were taken from the experiment. Animals treated with Pazopanib (31 days; median) and Sorafenib (29 days) displayed delayed tumor growth and survived significantly longer than control animals (22 days).

## DISCUSSION

Medulloblastoma is the most common paediatric brain tumor. Despite continuous optimization of intensive treatment regimens comprising surgery, radiation and chemotherapy, the prognosis for patients with high risk or relapsed disease remains grim [1-2]. Therefore, novel anti-neoplastic strategies with a more directed mode of action complementing standard cytotoxic therapy need to be explored. In this context, targeted therapeutics inhibiting multiple tyrosine kinases are considered promising. The MKI Pazopanib has recently shown good activity and tolerability in clinical trials against various tumor entities [10-14]. In adults Pazopanib does not only inhibit angiogenesis via inhibition of the VEGFR pathway but targets various oncogenic kinases known to be directly involved in medulloblastoma formation [3, 5-8]. Yet to date there are no reports assessing the potential of Pazopanib for medulloblastoma treatment.

Here we document for the first time the anti-carcinogenic potential of Pazopanib in paediatric medulloblastoma cell lines with differential molecular genetic group affiliations. The cell lines MEB-Med-8a, D283 Med and D341 Med display *c-myc* amplification and isochromosome 17, distinct characteristics of the most aggressive medulloblastoma subgroup 3, while Daoy cells show markers of SHH-group tumors [25-29]. Since Pazopanib shares a similar target profile with the MKI Sorafenib, yet there are pharmacodynamic and pharmacokinetic differences between these drugs that result in differential cytotoxic activity in various malignant and non-malignant tissues we evaluated both compounds in parallel [20, 23-24]. Of note, our evaluation of Pazopanib and Sorafenib efficacy delineates MKI-mediated inhibitory effects in medulloblastoma not only in a growth factor-deprived setting as previously demonstrated for Sorafenib by Yang et al. but also under standard growth conditions and in an orthotopic xenograft mouse model for both drugs [19]. At clinically relevant concentrations, reduction in viable cells is profound following treatment with Pazopanib and Sorafenib with differential modulation of cell proliferation and apoptosis in individual cell lines [21-22]. Thus, the anti-proliferative activity of Pazopanib and Sorafenib compares well for the cell line MEB-Med-8A and D283 with superior activity of Sorafenib in Daoy. None of the MKI induce significant proliferation arrest in D341 cells. Regarding apoptosis, both compounds induce cell death in all 4 investigated cell lines with a delayed response in Daoy and D341 cells. The putative decline in apoptosis rates over time as observed for the cell lines MEB-Med-8A and D283 Med might be due to chemoresistant subpopulations and drug degradation respectively. For the adherent cell lines Daoy and MEB-Med-8A cell cycle analysis and colony formation assays support our observations mentioned above by revealing that both inhibitors profoundly compromise clonogenicity and accumulate medulloblastoma cells irreversibly in S-Phase. For Sorafenib it has previously been shown that S-phase arrest is due to downregulation of cyclin D and E expression [30]. Cell cycle arrest in S-phase is suggested to increase the susceptibility of tumors to etoposides and cisplatine, chemotherapeutics that are often administered to medulloblastoma patients [31-32]. Moreover, combinations of these chemotherapeutic agents with MKI have either been proven successful or are momentarily under investigation in clinical trials for other cancers [33-34].

The differential susceptibility of the cell lines to MKI treatment noted above might be due to heterogeneous target expression. Indeed, this hypothesis is supported by our transcriptome analysis of VEGFR 1-3, PDGFR alpha/beta and c-kit expression in the cell lines (data not shown). While the VEGFR family and PDGFR beta are expressed equally on all cell lines, the expression of PDGFR alpha is only elevated in the responsive cell lines MEB-Med-8a, D283 and Daoy. Furthermore, MEB-Med-8a and D283 the most susceptible cell lines in our analysis display additionally high expression levels of c-kit. Of note, since Pazopanib and Sorafenib show similar affinities to the analysed targets most likely kinases apart from the investigated are responsible for the disparity between the two compounds.

Tumor cell migration is critical for invasion and metastasis. Our study complements previous published findings that document the anti-migratory capacity for Pazopanib and Sorafenib in adult cancer entities such as multiple myeloma [35-36]. For medulloblastoma we present first evidence that both compounds profoundly inhibit migration. In keeping with the observed attenuation of migration we detected a marked reduction in STAT3 phosphorylation suggesting that downstream of the MKI targets STAT3, as shown for numerous other cancer types, may be a mediator of cellular migration in medulloblastoma [37].

Beyond cellular mobility, STAT3 regulates several critical biological processes in tumorigenesis including cell-cycle progression, apoptosis, tumor angiogenesis and tumor-cell evasion of the immune system [38-39]. Recently, constitutive STAT3 activation has been documented in primary medulloblastoma tumor samples [19]. The activation exceeds that of other brain tumors examined such as glioblastomas, ependyomas and astrocytomas [40]. Moreover, STAT3 activation could be linked to chemoresistence of medulloblastoma cancer stem cells [41]. In keeping with these observations, we demonstrated that in the 4 investigated medulloblastoma cell lines, STAT3 protein is expressed and phosphorylated at TYR705.

Although Pazopanib has been proven successful in clinical trials in various neoplastic diseases, reports delineating the molecular mode of action are limited [10-14]. Previous published reports imply that the anti-carcinogenic properties of Pazopanib are due to inhibition of oncogenic kinases including VEGFR, PDGFR and c-kit [20, 42]. At present there is a great interest to identify downstream mediators of these kinases that may serve in future as biomarkers for the application of Pazopanib. Here we present the first evidence that Pazopanib similar to Sorafenib treatment inhibits STAT3 TYR705 phosphorylation. The observed suppression of STAT3 phosphorylation is in keeping with the observed proliferation arrest and induction of apoptosis in the analysed medulloblastoma cell lines [19, 43]. As the above mentioned receptor tyrosine kinases are known to mediate cellular response in part via STAT3 phosphorylation as the downstream effector, reduction in phosphorylated STAT3 is likely due to cumulative inhibition of these signalling pathways. In long-term analysis we demonstrated for the first time that Pazopanib and Sorafenib not only reduced STAT3 phosphorylation but also significantly decreased STAT3 protein levels in 3 of 4 medulloblastoma lines whilst levels of the housekeeping protein endoplasmatic reticulum-golgi intermediate compartment 53 (ERGIC53) and beta-tubulin respectively remained stable. Earlier studies limited their analysis of STAT3 phosphorylation to the first 24h of MKI treatment. At this early time point however, reduction in STAT3 protein levels could not be observed neither in our nor in the previously published studies [43-45]. Whether the long-term effects of Pazopanib and Sorafenib on STAT3 protein levels are medulloblastoma-specific or occur as well in other tumor entities will require further evaluation. In spite of equal protein loading, in the cell lines D283 and MEB-Med-8A, expression of the cytoskeleton protein beta tubulin was also reduced under the influence of Sorafenib with a lesser effect of Pazopanib. As interference of Sorafenib or Pazopanib with microtubular components and therefore spindle formation could arrest tumor cells in S-Phase, this finding is therefore in line with the cell cycle arrest following Sorafenib or Pazopanib treatment. Moreover, as a phenotypic correlate to cytoskeletal derangements we observed cell morphology changes with cell rounding and loss of adherent properties following Pazopanib and Sorafenib exposure (data not shown). The difference of Pazopanib and Sorafenib regarding their effect on cellular protein levels might be due to variations in the target profile of these MKI.

Here we present the first evidence that Pazopanib and Sorafenib show marked anti-neoplastic activity in an orthotopic xenograft mouse model of human medulloblastoma. Humanized orthotopic xenograft mouse models are key to preclinical drug evaluation since tumor growth and drug efficacy are analysed in context of the tumor-specific micro-environment and the biodistribution of the drug itself [46]. This is of particular interest when investigating brain tumors, in which biodistribution of the drug is crucial. Hence, to allow for recovery of the blood barrier, we delayed drug treatment for 1 week after medulloblastoma-instillation. Such “tumor growth delay” studies also mimic the clinical situation of pre-established tumor more adequately and are of stronger evidence in drug testing than the less stringent “tumor inhibition” studies based on concomitant tumor and drug inoculation [47]. Still we observed a significant reduction in tumor growth after two weeks and proofed for the first time that Pazopanib and Sorafenib significantly prolonged the survival of mice bearing intracranial human medulloblastoma. For *in vivo* analysis of Pazopanib and Sorafenib efficacy we chose MEB-Med-8a a patient-derived human medulloblastoma cell line that displays distinct molecular and genetic characteristics of the most aggressive *c-myc* amplified medulloblastoma of group 3. MEB-Med-8A derived tumors also mimic the clinical presentation of this medulloblastoma variant by showing large cell anaplastic histology and a rapid invasive growth pattern that leads to animal death within 2-3 weeks [48].

Although our *in vivo* model resembles group 3 medulloblastoma, we assume based on our *in vitro* findings and the common feature of medulloblastoma to express MKI targets, that SHH-, WNT- and group 4 medulloblastoma might also benefit from Pazopanib and Sorafenib treatment [3]. The expression profile of MKI target structures might therefore serve as future biomarker to incorporate Pazopanib and Sorafenib into the standard treatment regime for paediatric medulloblastoma.

In medulloblastoma, Pazopanib exhibits potent anti-neoplastic activity similar to the *in vitro and in vivo,* capacity of Sorafenib. In view of the favourable toxicity profile with regards to hematopoietic suppression together our data identify Pazopanib as a new promising candidate for targeted medulloblastoma therapy and provides a rational to progress to clinical evaluation of Pazopanib in combination with standard therapy for the treatment of paediatric medulloblastoma.

## MATERIAL AND METHODS

### Reagents and antibodies

Pazopanib (GW786034B) and Sorafenib (BAY 43-9006) were obtained from LC Laboratories. The primary antibody ERGIC53 was obtained form Santa Cruz and others primary antibodies pSTAT3 (TYR705, D3A7), STAT3 (124H6), beta-Tubulin, GAPDH were purchased from Cell Signalling while secondary antibodies were purchased from Dianova. Carboxyfluoreszein-Succinimidyl Ester (CFSE) was purchased from Invitrogen, while Hoechst 33342 was provided by Sigma. D-Luciferin Sodium Salt was purchased from PJK GmbH.

### Animals

Immunocompromised (NOD/SCID IL2Rγ^Null^ or NSG) mice were obtained from Charles Rivers (UK).

### Cell culture

The human medulloblastoma cell lines, Daoy (HTB 186), D283 Med (HTB-185) and D431 Med (HTB-187) were obtained from American Type Culture Collection (ATCC). The medulloblastoma cell line, MEB-Med-8A, was generated by Prof. T. Pietsch. The medulloblastoma cell lines Daoy, D283 Med and MEB-Med-8A were maintained in complete medium, namely Dulbecco's Modified Eagle Medium (DMEM, PAA) with L-glutamine supplemented with 1 mM sodium pyruvate (PAA), 1% penicilline/streptomycine (Invitrogen) and 10% fetal bovine serum (FBS, Invitrogen). The medulloblastoma cell line D341 Med was maintained in DMEM with L-glutamine supplemented with 1mM sodium pyruvate, 1% penicilline/streptomycine and 10% Human Serum (HS, PAA).

### Cell viability assay

Cell viability was assessed with CellTiter 96 Aqueous One Solution Cell proliferation Assay (Promega) which contains 3-(4,5-dimethylthiazol-2-yl)-5-(3- carboxymethoxyphenyl)-2-(4-sulfophenyl)-2H-tetrazolium (MTS). To ensure a linear growth curve over 48h for assessment of MKI-mediated effects, each well of 96-well plates was seeded with 2,5×10^3^ Daoy, 6×10^3^ MEB-Med-8A, 10^4^ D283 Med and 10^4^ D341 Med cells respectively. After overnight culture in complete medium, the cells were treated with increasing MKI concentrations. The vehicle Dimethylsulfoxid (DMSO) served as control. After 48h of MKI treatment, MTS was added according to the supplier's protocol and the absorbance was measured at 490 nm using an ELISA plate reader (Victor^2^ Wallac, Perkin Elmer). Cell viability was calculated in percent of control.

### Combined cell proliferation and apoptosis assay

Medulloblastoma cells were stained with CFSE according to the supplier's instructions. Daoy (3×10^5^/well), MEB-Med8A (5×10^5^/well) D283 Med (5×10^5^/well) and D341 Med (5×10^5^/well) cells were seeded in 6-well cell culture dishes in complete medium. After overnight culture, the cells were treated with MKIs at concentrations corresponding to patient plasma levels for a 24h, 48h or 72h period. Thereafter floating and attached cells were collected and stained with 7-AAD and Annexin V-Antibody (Annexin V-PE Detection Kit I, BD Bioscience) and analysed by flow cytometry (Navious, Beckman Coulter). Proliferation was traced by CFSE staining and apoptosis was detected by combined 7AAD/Annexin V staining and calculated in percent of control.

### Cell migration assay

For the *in vitro* scratch assay Daoy (5×10^5^/well) cells were plated in 12-well cell culture dishes. The cells were allowed to adhere and spread for 12h at 37 °C. The confluent monolayer was scratched in a straight line with a p200 pipette tip. The debris was removed and the cells were then incubated with Pazopanib and Sorafenib respectively. The vehicle DMSO served as control. After 12h and 24h of treatment, migration of cells into the “wound” was photographed at 10x magnification (Nikon Eclipse TiS inverted microscope attached to a CCD monochrome camera DS 2M). The distance of migration was analyzed by means of NIS-Elements Imaging Software.

### Cell cycle analysis

Daoy (2×10^5^/well) and MEB-Med-8A (3×10^5^ / well) cells respectively were plated in 6-well cell culture dishes. After 48h of treatment with the respective MKI the cells were exposed to 16 nM Hoechst 33342 and incubated for 45min at 37°C. Both floating and attached cells were harvested and analyzed by flow cytometry (Navious, Beckman Coulter). Dead cells were stained by Propidium Iodid (PI). After gating on live cells, single cells were gated using width and area parameters from Hoechst 33342. The area parameter histogram was used to determine the percentage of cells in G_1_, S and G_2_M phases.

### Immunoblotting analysis

A total protein concentration of 25 μg derived from medulloblastoma cell lines was separated by SDS-polyacrylamide gel electrophoresis and transferred to nitrocellulose membranes (BioRad). The membranes were blocked for 1h at RT in 1x Tris-buffered saline containing 0.1% tween-20 (TBST) supplemented with 5% BSA. Thereafter, the membranes were incubated with the primary antibodies (1/1000) overnight at 4°C and subsequently with the respective secondary antibody (1/10000) for 1h at room temperature. Immunoreactivity was detected by chemiluminescence and quantified by means of a ChemiDoc XRS Imaging System (Bio-Rad).

### Colony formation assay

The cell lines Daoy (200 cells/well) and MEB-Med-8A (1000 cells/well) were plated in six well cell culture dishes. The cells were allowed to adhere and spread properly for 12h at 37 °C. Thereafter the cells were exposed to 1 and 10 μM of GDC-0941 respectively for. After 48h of exposure the cells were washed with standard medium to remove any trace of the inhibitor and cultured for another week. Colony numbers, colony size and the fraction of surface covered with colonies was assessed by IMAGEJ. Particles smaller than 20 pixel^2^ were excluded from the analysis since these represented mainly stain artefacts, cell detritus or non-proliferating single cells.

### Lentiviral particles and stable cell lines

Lentivirale particle were generated by co-transfection of HEK293T Lenti-X cells (Clontech) with packaging plasmids (pMD2.G, pMDLg/pRRE, pRSV-REV, Addgene) and the lentiviral transfervector (pLenti-III-UbC-Luc2, Applied Biological Materials Inc.) expressing Luciferase 2. After 48 and 72 hours the supernatants were pooled, filtered through a 45 um filter and ultra centrifuged at 32000 rpm 4°C for 1h. The virus titers were determined by HIV p24 antigen test (Elecsys, Roche Diagnostics GmbH). MEB-Med-8A cells were transduced with virus particles (MOI 5) and thereafter selected with puromycin for cell clones that show stable luciferase expression (Invitrogen).

### Orthotopic transplantation and tumor formation

Immunocompromised NSG mice were used for transplantation. The animals were bred and housed in a specific pathogen-free animal facility at the house for experimental therapy of the university of Bonn. All experiments were conducted according to protocols approved by the institutional animal use and care committee of North Rhine Westphalia (Germany). To establish intracranial tumors, medulloblastoma cells were resuspended in PBS and injected perpendicular to the cranial surface via 5 μL 65/70 mm; pst2 Hamilton 7105N syringe into the right cerebellar hemisphere (1 mm to the right of the midline, 1 mm posterior to the coronal suture, and 3 mm deep) of 5-9 week old anesthetized NSG mice. During the procedure the mice were fixated using a stereotaxic frame with a mouse adaptor. After transplantation the animals were monitored daily and sacrificed when symptoms of tumor growth occurred. All procedures were in strict accordance with the University of Bonn Medical Center Policy on the Use and Care of Laboratory Animals (University of Bonn Medical Center Policy and Welfare Committee, Document ID: 87-51.04.2011.A033).

### *In vivo* bioluminescent imaging

For the bioluminescent imaging the mice were anesthetized via a ketamine/xylazine combination. Thereafter the animals were injected intraperitoneal with 125 mg/kg of D-luciferin. 15 min after the injection the animals were imaged using a IVIS 200 imaging station (Caliper Life Sciences). Regions of interest were defined using living image software, and the total photons/s/sr/cm^2^ (photons per second per steradian per square cm) were recorded weekly to monitor tumor growth and therapy response. To determine the growth rate of the tumor the gain in bioluminescence per week was calculated.

### *In vivo* inhibitor treatment

To study effects of the MKIs Pazopanib and Sorafenib on tumor growth *in vivo* we transplanted 2×10^4^ MEB-Med-8A cells into the cerebellum of NSG mice. Seven days after transplantation the mice were randomly separated into two groups. Group 1 was given the vehicle EL-ethanol (50:50; Sigma cremophor EL, 95% ethyl alcohol) while group 2 was exposed to 60 mg/kg Pazopanib and the Group 3 to 30 mg/kg of Sorafenib. The drugs were administered once daily by oral gauge. Survival was defined as the time from transplantation until symptom onset.

### Statistical analysis

The two-sided Student's *t*-test was applied to determine statistical significance between groups. p<0.05 (*), was considered as statistically significant. Values stated within text and figures represent mean ± standard deviation.

## Abbreviations

ATCC: American Type Culture Collection
mAb: Monoclonal antibody
CFSE: Carboxyfluoreszein-Succinimidyl Ester
CNS: Central nervous system
DMEM: Dulbecco's Modified Eagle Medium
DMSO: Dimethylsulfoxid
ERGIC53: Endoplasmatic reticulum-golgi intermediate compartment 53 kDa protein
FBS: Fetal bovine serum
h: Hour
HS: Human Serum
M: Molar
m: Meter
MELK: maternal embryonic leucine zipper kinase
MKI: Multi-kinase Inhibitor
MTS: 3-(4,5-dimethylthiazol-2-yl)-5-(3-carboxy-methoxyphenyl)-2-(4-sulfophenyl)-2H-tetrayolium
min: Minute
PDGFR: Platelet-derived growth factor receptor
PI3K: Phosphoinositid-3-kinase 3
STAT3: Signal transducer and activator of transcription 3
TKI: Tyrosine kinase inhibitor
TYR: Tyrosine
VEGFR: Vascular growth factor receptor
